# Does peak expiratory flow measured sitting differ from that measured standing? A cross-over study in primary care in Barbados

**DOI:** 10.3399/bjgpopen18X101592

**Published:** 2018-06-27

**Authors:** O Peter Adams, Khatija AS Mangera, Ian R Hambleton, Euclid H Morris, Joanne L Paul-Charles

**Affiliations:** 1 Dean, Faculty of Medical Sciences, University of the West Indies Cave Hill Campus, St Michael, Barbados; 2 Registrar, General Practice Unit, Edgar Cochrane Polyclinic, Wildey, St. Michael, Barbados; 3 Professor in Biostatistics, George Alleyne Chronic Disease Research Centre, The Caribbean Institute for Health Research, The University of the West Indies, Bridgetown, Barbados; 4 Lecturer in Family Medicine, Faculty of Medical Sciences, University of the West Indies, Cave Hill Campus, St Michael, Barbados; 5 Lecturer in Family Medicine, Faculty of Medical Sciences, University of the West Indies, Cave Hill Campus, St Michael, Barbados

**Keywords:** Asthma, peak expiratory flow, peak expiratory flow meter, technique, primary care, general practice

## Abstract

**Background:**

Several authorities recommend measuring peak expiratory flow (PEF) standing. Limited evidence suggests that PEF obtained sitting is similar in magnitude but there are no studies in African populations.

**Aim:**

To determine in adults aged 18–60 years if PEF measured sitting differs from that measured standing.

**Design & setting:**

Crossover design with alternating position of initial measurement in people attending primary care clinics in Barbados.

**Method:**

Quota sampling by age, sex, and clinic of adults aged 18–60 years was done and an interviewer-administered questionnaire was completed. PEF sitting and standing was measured with an European Union (EU) scale Mini-Wright® meter. The highest of three readings in each position was used and the difference in means tested for significance using the paired sample *t*-test.

**Results:**

Characteristics of the 199 participants were 44% male; 96.5% of African descent; mean age 37 years (standard deviation [SD] 12.8); 22% with an asthma diagnosis; 23% tobacco users; and 22% marijuana users. Mean PEF standing was 438.4 versus 429.7 lmin^–1^ sitting, mean difference 8.7 (95% confidence interval [CI] = 3.6 to 13.8). For men, mean PEF standing was 518.7 versus 506.3 lmin^–1^ sitting, mean difference 12.4 (95% CI = 3.3 to 21.5). For women, mean PEF was 374.7 standing versus 368.9 lmin^–1^ sitting, mean difference 5.8 (95% CI = 0.11 to 11.5). A Bland-Altman plot accounting for trend and a Lin’s correlation coefficient of 0.935 demonstrated good agreement between standing and sitting PEF.

**Conclusion:**

PEF measurements are reduced when performed sitting compared to standing. The difference is small and unlikely to alter clinical management in most cases.

## How this fits in

PEF readings are used to help guide the management of asthma. Most authorities recommend that measurement be done standing but limited available evidence suggests that readings in the sitting position are similar in magnitude. In this study, PEF readings are reduced on average by 8.7 lmin-1 (95% CI = 3.6 to 13.8) when performed sitting compared to standing. This difference is unlikely to alter clinical management.

## Introduction

About 334 million people worldwide have asthma, with about 14% of children and 8.6% of adults aged 18–45 years experiencing symptoms.^1^

Patients and physicians do not always accurately recognise asthma symptoms or their severity. It is recommended that lung function measurements such as the PEF be included in both self and physician assessment.^2^ PEF is the maximum flow achieved during an expiration delivered with maximal force starting from the level of maximal lung inflation.^3^ It is measured with a PEF meter that can be used in the home, clinic, and hospital setting to evaluate severity of airflow limitation and the response to treatment, and help guide therapeutic decisions.

In order to obtain accurate and reproducible PEF readings, a standardised technique should be followed. Several authorities recommend that measurements be done while standing. These authorities include the Global Initiative for Asthma (GINA),^4^ American Lung Association,^5^ American Academy of Allergy Asthma and Immunology,^6^ National Asthma Education and Prevention Program (NAEPP),^7^ the National Institutes of Health Medline Plus,^8^ and the Caribbean Health Research Council.^2^ However, the British guideline on the management of asthma recommends that the patient can be sitting or standing.^9^ Further predicted peak flow values for adult that accompany two commonly used PEF meters (Mini-Wright and TruZone®)^3,10^ are based on a study by Nunn and Gregg which does not specify the position in which the measurements were done.^11^ These values were derived from a white European population.

In a busy primary care setting, and especially with patients who may have difficulty standing, it may be quicker and more convenient to do the measurement sitting rather than standing. To the authors' knowledge, only three published studies^12–14^ have addressed the question of whether it matters if PEF is measured sitting upright in a chair or standing; the first study of 33 healthy people (39% white and 61% Asian American, aged 18–58 years) found no difference in PEF (mean PEF 461 lmin^–1^ sitting versus 469 lmin^–1^ standing, *P*>0.1).^12^ The second study was done on 211 healthy College of Pharmacy students (aged 20–43 years) from the University of Tennessee of various but unspecified ethnicities; the university is, however, 82% white.^15^ The study found that PEF did not differ by position (506 lmin^–1^ sitting versus 508 lmin^–1^ standing, *P* = 0.45).^13^ The third study was done in white European brass players with a mean age of 21 years attending music and drama colleges in Wales. This study used spirometry rather than a PEF meter and did not find a statistical difference in percentage of predicted PEF achieved (85.6 ± 1.76% sitting upright versus 88.0 ± 1.94% standing, *P* = 0.117).^14^ None of the studies were carried out in a population of predominantly African-descent. It has been recognised that in addition to age, sex, and height that there may be difference in lung function between people of different ethnicities.^16–17^

While none of the studies showed a statistical difference in PEF, the findings may not be generalisable to adults of all ages and ethnicities. None of the previous studies were performed among patients in a primary care setting, a situation where PEF is commonly measured to guide decisions with regards clinical care. The objective of this study is to determine whether a difference between sitting and standing PEF exists in the Barbadian population aged 18–60 years, 92% of whom are of African origin.^18^

## Method

### Setting

Barbados, an island in the Eastern Caribbean has a population of approximately 280 000.^18^ Barbadians have a choice between comprehensive free public primary health care and fee-for-service private care. Public sector primary care, funded through taxation and organised by the Ministry of Health, is provided through nine polyclinics strategically located across the island. Each polyclinic serves people from a catchment geographic area and provides multidisciplinary services. This study was conducted in four of these polyclinics. The polyclinics chosen spanned rural and urban areas and included two where study authors worked.

### Study design and participants

This study had a crossover (within-subjects) design and was conducted on people attending four of the eight public sector polyclinics in the Barbados. PEF meters are often used in the management of asthma in this setting. Quota sampling was used to achieve an approximately equal distribution by sex, by age group (18–30 years, 31–44 years, and 45–60 years), and by polyclinic. Inclusion criteria were that participants should be aged 18–60 years, and be a patient or accompanying person in the waiting room of the clinic. Exclusion criteria were the presence of a respiratory tract infection, cough, wheezing, febrile illness, pregnancy, and inability to follow verbal instructions.

### Sample size

A previous study reported SDs for PEF readings of 60 lmin^–1^ for women and 71 lmin^–1^ for men.^13^ Assuming a PEF SD of 71 lmin^–1^ and a correlation between sitting and standing PEF of 0.5, a sample size of 101 was needed to be 95% certain that a difference of 20 lmin^–1^ between sitting and standing PEF was not due to chance, and to have an 80% power to detect such a difference.

### Recruitment

All patients and accompanying persons in the waiting room of the clinic on data collection days were given a brief description of the study. Those considered initially eligible were taken to a private area, eligibility was confirmed, and informed consent obtained. Participants were given the option of not signing the consent form and giving verbal consent only since information on marijuana use, an illegal activity, was being collected. Data collection forms were labelled with a code, and kept separately and securely from the consent forms.

### Questionnaire and examination

An interviewer-administered questionnaire collected demographic data including age, ethnicity, sex, and medical conditions and habits that may affect lung function including asthma, tobacco use, marijuana use, and inhaled medication use. The study was piloted on six participants. A small adjustment was made to the questionnaire and these participants were not included in the study.

### PEF measurement

A Mini-Wright (standard range) EU scale PEF meter (part reference: 3103387) designed for multiple patient uses and one-way valve disposable mouthpieces were used.

Fieldworkers were trained on the use of the PEF meter and on the study protocol. The first participant performed the PEF measurement sitting and then standing. With each subsequent participant, the order of the starting position (sitting or standing) was alternated. After one to three trial attempts to familiarise participants with PEF meter use, the following instructions based on the NAEPP^7^ and the American Lung Association were followed:^5^

1. Sit with back straight in a chair and feet flat on the floor, or stand straight.
2. Remove any food or gum from your mouth.
3. Make sure the marker on the peak flow meter is at the bottom of the scale.
4. Breathe in slowly and deeply. Hold that breath.
5. Place the mouthpiece on your tongue and close lips around it to form a tight seal (do not put tongue in the hole).
6. Blow out as hard and fast as possible. Blow a 'fast hard blast' rather than 'slowly blowing' until nearly all of the air has been emptied from the lungs.
7. If the participant coughs or makes a mistake, discard reading and do it over again.
8. Repeat two more times.
9. Rest briefly then repeat in alternate position —﻿ standing or sitting.

Participants were specifically asked to place the mouth piece well into the mouth to avoid accelerating air with the tongue.^19^

### Analysis

After examining PEF for distributional normality, the mean difference in PEF between sitting and standing positions was calculated and formal statistical significance examined using the paired sample *t*-test. The Pearson’s correlation coefficient (precision) and the bias correction factor (a measure of how far each PEF observation deviates from the 45° degree line through the origin) were calculated and their product was used to estimate Lin’s concordance coefficient, a measure of agreement. A Bland-Altman plot accounting for trend was used to demonstrate agreement between standing and sitting PEF through the whole range of values. The influence of selected patient characteristics on mean PEF was explored using linear regression, including age, asthma diagnosis, height, body mass index (BMI), and sex as potential model predictors. For all formal comparisons, statistical significance was assumed at the 5% level, with exact *P*-values and 95% CIs presented at all times to clarify the extent of any relationship. All analyses used the Stata statistical software (release 15).

## Results

Of the 209 people approached, eight were deemed ineligible due to a respiratory tract infection or wheezing. One person declined to participate and complete data was not obtained for one person. Complete PEF data was available on 199 people and these were included in the analysis. The sample consisted of 88 (44%) men and 111 (56%) women. The mean age was 37.2 years (SD 12.8), and the population was 96% of African descent; 22% had asthma, 23% were current tobacco users, and 22% were marijuana users (Table 1). All the people who reported a diagnosis of asthma reported using prescription inhalers, with 32% reporting beta agonist use alone and 68% beta agonist plus steroid use.

**Table 1.** Demographic characteristics of study participants

	Total	Men	Women
Sex, *n* %	199	88 (44.2)	111 (55.8)
**Ethnicity, *n* (%)**			
African descent	192 (96.5)	86 (97.7)	106 (95.5)
Mixed	6 (3.0)	1 (1.1)	5 (4.5)
East Indian	1 (0.5)	1 (1.1)	0 (0)
Mean age, years (SD)	37.2 (12.8)	37.9 (13.1)	36.7 (12.6)
Asthma diagnosis, *n* (%)	44 (22.1)	14 (15.9)	30 (27.0)
Mean height, cm (SD), *N *= 197	168.5 (9.8)	174.7 (7.2)	163.6 (8.8)
BMI, kg/m^2^ (SD), *N *= 195	28.2 (7.4)	26.1 (6.3)	29.8 (7.7)
Current tobacco user, *n* (%)	29 (23.4)	20 (29.8)	9 (15.8)
Marijuana use in last month, *n* (%)	44 (22.2)	29 (33.3)	15 (13.5)

BMI = body mass index. SD = standard deviation.

Mean PEF was 8.7 lmin^–1^ (95% CI = 3.6 to 13.8) higher in the standing compared to the sitting position. For men, the difference was 12.4 lmin^–1^ (95% CI = 3.3 to 21.5) and women 5.8 (95% CI = 0.11 to 11.5) (Table 2). There was no difference between sitting and standing PEF for people who reported a diagnosis of asthma.

**Table 2.** Mean peak expiratory flow rate measurements sitting and standing

	Standing PEF lmin^–1^ (SD)	Sitting PEF (SD)	Difference (95% CI)	*P-*value
Total (*n* = 199)	438.4 (121)	429.7 (118)	8.7 (3.6 to 13.8)	0.001
Men (*n* = 88)	518.7 (111)	506.3 (107)	12.4 (3.3 to 21.5)	0.008
Women (*n* = 111)	374.7 (86)	368.9 (86)	5.8 (0.11 to 11.5)	0.046
Asthma diagnosis (*n* = 44)	395.3 (115)	393.0 (103)	2.4 (-6.7 to 11.5)	0.600
No asthma (*n* = 153)	451.6 (121)	441.2 (120)	10.4 (4.3 to 16.5)	0.001

P<0.05 is signficant. PEF = peak expiratory flow. SD = standard deviation.

**Figure 1.**
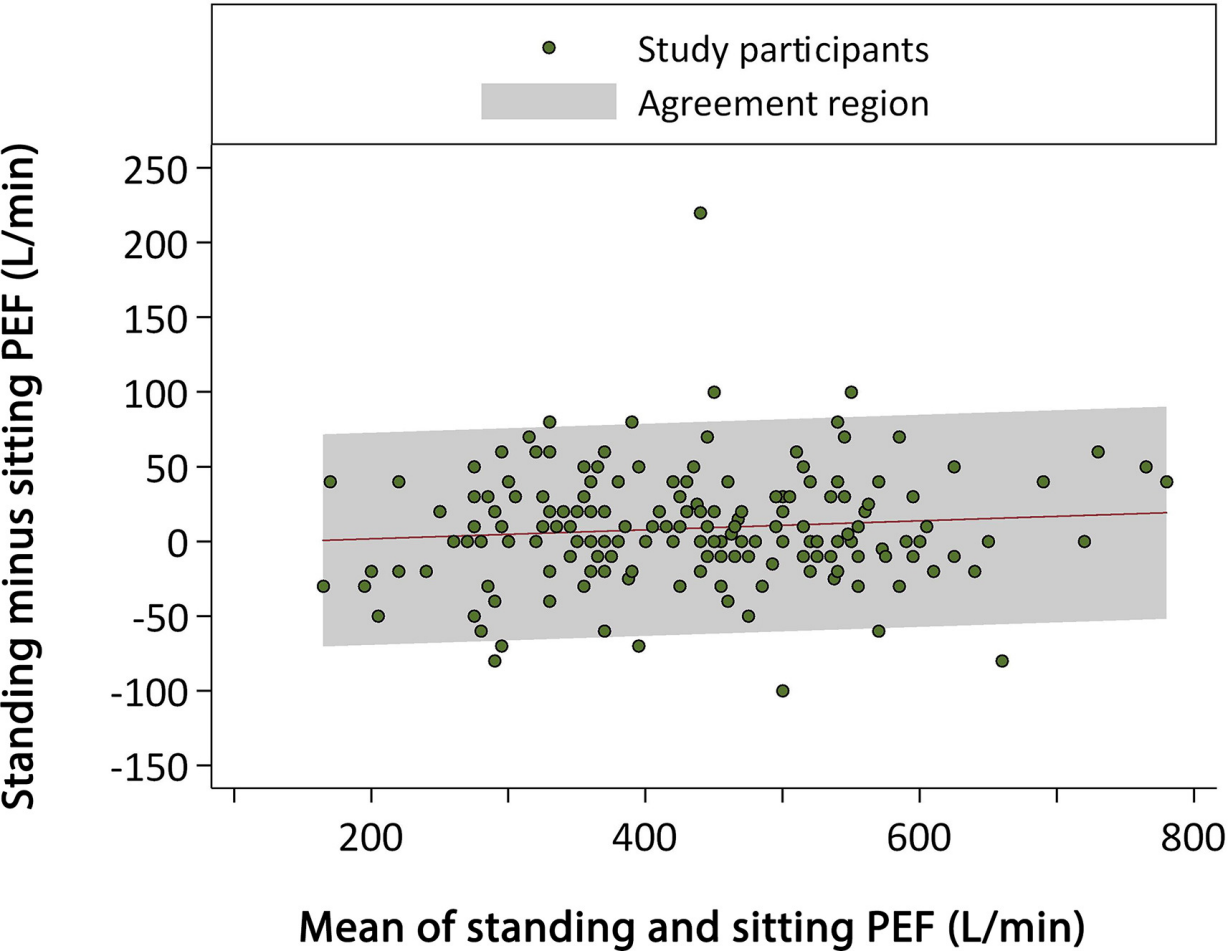
Bland-Altman plot of standing versus sitting peak expiratory flow (PEF).

Agreement between readings done in the sitting and standing positions was good, with a Lin’s correlation coefficient of 0.95 (95% CI = 0.94 to 0.96) (Table 3). The SD of the distribution of the difference in standing and sitting PEF values was 36.4 lmin^–1^. A Bland-Altman plot accounting for trend (Figure 1) showed good agreement between standing and sitting PEF measurements except that the mean standing values were on average 8.7 lmin^–1^ higher than the sitting values. The agreement appeared to be maintained for the whole range of PEF values.

**Table 3.** Correlation and agreement between measurement of PEF when standing and when sitting

	Women	Men	All
Pearson’s correlation coefficient	0.94	0.92	0.95
Bias between readings (slope, intercept)	1.00 (1.00, 5.68)	0.99 (1.04, −6.11)	1.00 (1.03, −4.12)
Agreement^*a*^ (95% CI)	0.94 (0.912 to 0.959)	0.92 (0.88 to 0.95)	0.95 (0.938 to 0.964)
Limits of agreement (95% CI)	5.81 (−53.60 to 65.22)	12.39 (−71.51 to 96.28)	8.72 (−62.66 to 80.10)

^*a*^Lin’s concordance coefficient (Pearson’s correlation coefficient x bias between readings). PEF = peak expiratory flow.

The mean PEF of men was higher than women in both the sitting (mean difference 137 lmin^–1^, 95% CI = 110 to 164, *P*<0.001) and standing (mean difference 144 lmin^–1^, 95% CI = 116 to 171, *P*<0.001) positions, and was also higher for non-asthmatics compared to those with asthma in both the sitting (mean difference 48, 95% CI = 9 to 88, *P* = 0.02) and the standing (mean difference 56, 95% CI 16 to 97, *P *= 0.01) positions. After adjusting for age, height, and BMI, sex and asthma remained statistically significant predictors of standing PEF (sex: men higher than women, mean difference 114 lmin^–1^, 95% CI = 81 to 148, *P*<0.001; asthma: PEF higher among those without asthma, mean difference 33 lmin^–1^, 95% CI = 1 to 67, *P *= 0.05). Using the same adjusting factors, only sex remained a statistically significant predictor of sitting PEF (sex: men higher than women, mean difference 109 lmin^–1^, 95% CI = 77 to 142, *P*<0.001; asthma: PEF higher among those without asthma, mean difference 26 lmin^–1^, 95% CI = −7 to 59, *P *= 0.12). Adjusted models explained between 36 and 37% of data variation.

## Discussion

### Summary

This study found that in a population largely of African descent, PEF measured standing is on average higher than that measured sitting. However, the difference is small and may not make a difference to clinical management. Furthermore, for people with a history of asthma the difference in PEF was not significantly different by position of measurement. Agreement between readings done in the sitting and standing positions was good over the whole range of PEF values. Linear regression indicated that sex was a significant predictor of PEF in both the standing and sitting positions after adjusting for age, height, and BMI.

### Strengths and limitations

Strengths of this study were that participants were selected over a wide age range, were typical of those attending primary care, and that bias was reduced by alternating the initial position of measurement. A limitation of this study is that it did not include people who were wheezing and therefore may not be generalisable to this situation. It was decided not include such people to avoid subjecting them to a study protocol that was unnecessary for their treatment and that might cause discomfort. However, the study did include people with asthma who were being prescribed medication and this is also a population in which PEF estimation is indicated in practice.

### Comparison with existing literature

All three previous published studies examining the effect of sitting upright versus standing that were identified have shown a small but statistically insignificant higher PEF when measured standing compared to sitting.^12–14^ None of the previous studies involved a population of predominantly African descent.

All 22% of the sample who reported a diagnosis of asthma also reported using prescription inhalers for the condition. A previous study in Barbados published 30 years ago estimated an asthma prevalence of 16% in children aged 13–14 years.^20^ Adult prevalence data for Barbados are not available. It is likely that people on prescription medicine for a chronic condition will be overrepresented in a clinic population.

### Implications for research and practice

The basis of the recommendation of several authorities to use the standing position as opposed to a sitting position when measuring PEF is not clear, and the often quoted normal adult PEF range is based on a study that did not specify the position used in the measurement.^10,11^ The present study therefore adds information that may be useful to practice guideline writers and for individual physicians making decisions in the clinical setting.

Performing the test sitting in a primary care setting would usually be more convenient and quicker for patients. A few patients, for example frail and older people, may have trouble standing and the impact of position on these subpopulations can be investigated by future research. In cases where standing is difficult, it may be reasonable to do the measurement sitting and note that the reading may underestimate that done in the standing position by about 9 lmin^–1^ or about 2%. To help comparison, the position in which the PEF is measured can be documented and future PEF measurements performed in the same position.
